# Cardiometabolic drugs after stroke: time to embrace SGLT2 inhibitors and incretin-based therapies?

**DOI:** 10.3389/fstro.2026.1918465

**Published:** 2026-08-25

**Authors:** Håkon Ihle-Hansen, Hege Ihle-Hansen, Rolf A. Blauenfeldt, Jesse Dawson, Søren Z. Diederichsen, Guri Hagberg

**Affiliations:** 1 Department of Medicine, Bærum Hospital, Vestre Viken Hospital Trust, Drammen, Norway; 2 Department of Acute Medicine, Oslo University Hospital, Ullevål, Oslo, Norway; 3 Danish Stroke Center, Department of Neurology, Aarhus University Hospital, Aarhus, Denmark; 4 Department of Clinical Medicine, Aarhus University, Aarhus, Denmark; 5 Institute of Cardiovascular and Medical Sciences, College of Medical, Veterinary and Life Sciences, University of Glasgow, Glasgow, United Kingdom; 6 Department of Cardiology, Copenhagen University Hospital - Rigshospitalet, Copenhagen, Denmark; 7 Department of Neurology, Oslo University Hospital, Ullevål, Oslo, Norway

**Keywords:** CKD, CVD, GLP-1 RA, heart failure, secondary prevention, SGLT2 inhibitors, stroke

## Abstract

Stroke is a multifactorial cardiovascular disease (CVD), which predominantly affects older adults. Consequently, cardiovascular risk factors and related comorbidities are highly prevalent among people presenting with acute stroke. These conditions, and the stroke event itself, may require specific pharmacological therapies to reduce subsequent risk. Over recent decades, two novel pharmacological drug classes, sodium–glucose cotransporter-2 inhibitors and glucagon-like peptide-1 receptor agonists, which were initially developed as glucose-lowering therapies for type 2 diabetes mellitus, have been used for management of related cardiovascular conditions. These agents are not currently included in secondary stroke prevention guidelines, despite growing evidence suggesting that they may directly or indirectly improve long-term, stroke-related outcomes. It is therefore essential that the stroke community remains up-to-date with these developments while awaiting updated clinical guidelines. This will ensure optimal management of coexisting cardiovascular disease and reduce subsequent risk. In this commentary, we summarize and discuss what we consider to be the most relevant and impactful recent studies for contemporary clinical stroke practice.

## Introduction

The American Stroke Association released its latest Secondary Stroke Prevention Guideline in 2021, while the European Stroke Organization released theirs in 2022 (Kleindorfer et al., 2021; Dawson et al., 2022). Both guidelines aim to reduce the risk of recurrent stroke and other major cardiovascular outcomes, recognizing that individuals with stroke are at increased risk of recurrent cerebrovascular and cardiovascular events. The guidelines are centered around the most common pharmacological modifiable cardiovascular risk factors and conditions, such as diabetes, blood lipids and hypertension.

However, since the publication of these guidelines, evidence concerning benefits on cardiovascular outcomes for two novel drug classes has grown substantially. Sodium–glucose cotransporter 2 (SGLT2) inhibitors and incretin-based therapies (glucagon-like peptide-1 receptor agonists GLP-1 RA) and glucose-dependent insulinotropic polypeptide (GIP) were initially developed for the treatment of type 2 diabetes mellitus (T2DM), but have been proven to have cardiovascular benefits extending beyond glycemic control. This has shifted the therapeutic paradigm, positioning these drugs as cardiometabolic agents with disease-modifying effects playing a central role in cardiovascular risk reduction.

Comprehensive post-stroke work-up often reveals previously undiagnosed cardiovascular comorbidities requiring action, particularly T2DM, impaired kidney function, and heart failure (HF). To optimize care and improve long-term outcomes, stroke practice may need to evolve ahead of guideline updates.

This commentary reviews the role of the SGLT2 inhibitors and incretin-based therapies in stroke survivors. The publications discussed in this commentary were selected based on the authors' clinical and scientific expertise, with emphasis on contemporary clinical practice guidelines, randomized controlled trials, and meta-analyses considered most relevant to the topic.

### Developed for the purpose of glycemic control

T2DM is a frequent comorbidity in stroke, whether pre-existing or newly diagnosed, affecting approximately one in five individuals with acute stroke. It is associated with both macrovascular and microvascular complications. However, unlike microvascular complications, macrovascular outcomes show a weaker and less direct association with glycemic control. Consequently, macrovascular complications such as stroke and other cardiovascular events have remained major long-term risks for patients with T2DM, irrespective of hemoglobin A1c (HbA1c) levels. Notably, before SGLT2 inhibitors and incretin-based therapies became available, the insulin-sensitizing agent pioglitazone had the most substantial evidence for potential cardiovascular benefit (Kernan et al., 2016). However, its side effects have limited its widespread use in clinical practice (Dawson et al., 2022).

Due to evidence that some older antidiabetic drugs were associated with adverse cardiovascular outcomes, the U.S. Food and Drug Administration mandated in 2008 that all new glucose-lowering agents demonstrated cardiovascular safety in outcomes trials. This requirement led to the discovery that certain drugs not only met safety criteria but also reduced cardiovascular events and conferred additional benefits for conditions such as HF and chronic kidney disease (CKD).

### Sodium–glucose cotransporter 2 inhibitors

SGLT2 inhibitors block the sodium–glucose cotransporter 2 in the proximal renal tubules, increasing urinary glucose and water excretion. This results in modest reductions in blood glucose, blood pressure, and body weight. It is generally well tolerated, but is not recommended if glomerular filtration rate (GFR) is < 20 mL/min/1.73 m^2^, and is associated with an increased risk of ketoacidosis and genital infections.

Despite only limited reductions in HbA1c, SGLT2 inhibitors significantly reduce cardiovascular events and improve survival in patients with T2DM at high cardiovascular risk. These benefits appear to be consistent across multiple agents (McGuire et al., 2021), supporting a class-wide effect and suggesting that people with stroke and concomitant T2DM should be considered for SGLT2 inhibitor therapy regardless of baseline glucose control or concomitant glucose-lowering treatment (Perkovic et al., 2019; Wiviott et al., 2019; Bhatt et al., 2021; Marx et al., 2023; Committee, 2024). Subgroup analyses indicate that the effects are similar in patients with prior stroke and for patients with frailty (Fitchett et al., 2019). The recently updated National Institute for Health and Care Excellence (NICE) guidelines recommend SGLT2 inhibitors as first-line therapy alongside metformin (National Institute for Health and Care Excellence, 2026).

The cardiovascular benefits of this drug class have been driven mainly by reductions in cardiovascular mortality and HF hospitalizations, with modest reduction in atherosclerotic cardiac events (Neal et al., 2017; Perkovic et al., 2019; Wiviott et al., 2019). Importantly, current evidence does not support a clear reduction in recurrent stroke with SGLT2 inhibitors therapy (Tsai et al., 2021).

**Figure 1:**
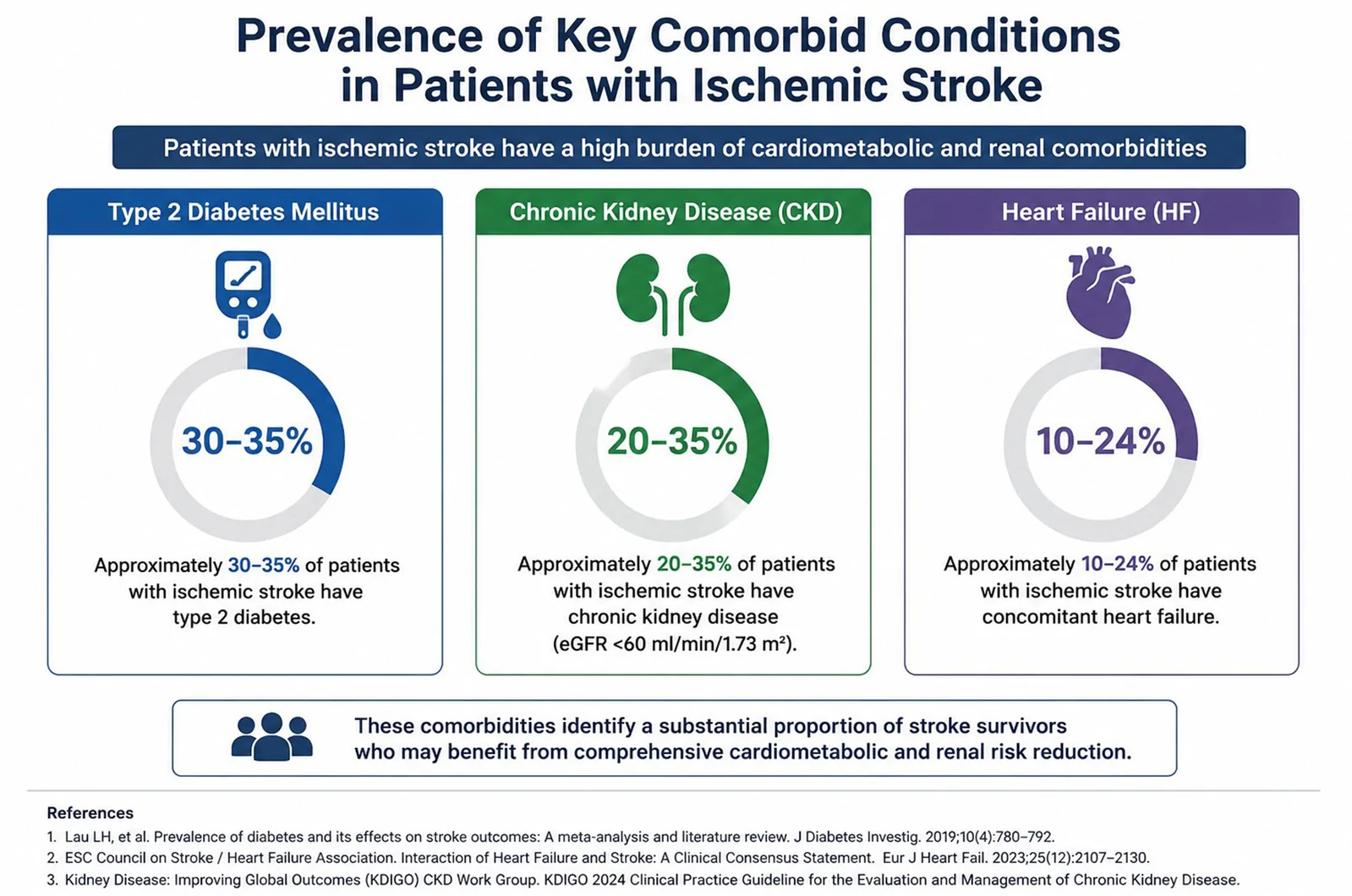
Approximate prevalence ranges of three common comorbidities in acute stroke: type 2 diabetes mellitus, heart failure and chronic kidney disease. Ranges are estimates and reflect variation by age, stroke subtype, and data source.

Subsequent trials have shown that SGLT2 inhibitors confer benefits in reducing HF-related hospitalizations regardless of whether ejection fraction is reduced (HFrEF) or preserved (HFpEF). In CKD, defined as chronic reduced GFR < 60mL/min/1.73 m^2^, they slow progression and preserve kidney function, even in the absence of T2DM. Heart failure and nephrology guidelines therefore recommend SGLT2 inhibitors as first line medical treatment in case CKD with albuminuria or HF, regardless of the presence T2DM (van der Aart-van der Beek et al., 2022; McDonagh et al., 2023; Levin et al., 2024). A reduced GFR and HF are common among people with stroke (Appelros et al., 2003; Rajesh et al., 2024) (Figure 1). It is therefore likely that a large number of people seen in stroke services might be eligible for SGLT2 inhibition.

### Incretin-based therapies

GLP-1 is secreted from intestinal L-cells, by pancreatic α-cells, and by neurons in nucleus of the solitary tract in response to food intake. GIP is secreted from intestinal K-cells and contributes to glucose homeostasis by enhancing glucose-dependent insulin secretion. These hormones play a key role in regulating digestion, appetite, and blood sugar through stimulation of insulin secretion, suppression of glucagon release, delayed gastric emptying, and promoting satiety (Zheng et al., 2024). GLP-1 RA and GIP drugs mimic the effects of endogenous hormones but have longer half-lives. GLP-1 RAs are generally well tolerated. The most common side effects are gastrointestinal, such as nausea.

Similar to SGLT2 inhibitors, incretin-based therapies have demonstrated benefits beyond improved glycemic control in patients with T2DM, including significant weight reduction and improvements in renal and HF outcomes (Kosiborod et al., 2024; Perkovic et al., 2024; Badve et al., 2025).

The substantial weight loss across incretins suggest a class effect (Aronne et al., 2025), with greater reduction from injectable than oral formulations (Wharton et al., 2025). Accordingly, recent American Gastroenterological Association guidelines recommend incretins, alongside life-style intervention, for adults with obesity regardless of T2DM, to reduce metabolic and obesity-related complications (Grunvald et al., 2022).

In patients with T2DM, the cardiovascular benefit is reflected in an approximately 14% reduction in the composite three-point major adverse cardiovascular event (MACE) endpoint (myocardial infarction, stroke, and cardiovascular death), corresponding to a number needed to treat of 65 in patients with T2DM to avoid one MACE event over 2.1 years (Marso et al., 2016; Sattar et al., 2021; Marx et al., 2022). When broken down by individual components, semaglutide reduced nonfatal stroke (HR 0.61; 95% CI 0.38–0.99) and showed a trend toward reduction in nonfatal myocardial infarction (HR 0.74; 95% CI 0.51–1.08), but had no effect on cardiovascular death (HR 0.98; 95% CI 0.65–1.48) (Marso et al., 2016). A meta-analysis of GLP-1 RA has confirmed a reduction in stroke risk (HR 0.83, 95% CI 0.76–0.92, *p* = 0.0002) and smaller effect on myocardial infarction in people with T2DM (HR 0.90, 95% CI 0.83–0.98, *p* = 0.02) (Sattar et al., 2021). A reduction in 90-day recurrent stroke was observed in a recent small study of patients with T2DM following minor stroke or high-risk TIA (Zhu et al., 2026). By contrast, SGLT2 inhibitors have not shown a comparable effect on stroke.

Based on current evidence, GLP-1 RAs share recommendations with SGLT2 inhibitors in patients with T2DM and cardiovascular disease (CVD) or high cardiovascular risk, and are preferred in those who are overweight or obese (ElSayed et al., 2022). Combination therapy of SGLT2 inhibitors and GLP-1 RA has not been evaluated in dedicated randomized controlled trials, but a meta-analysis of the current evidence suggest it is safe and effective (Mousavi et al., 2025). Despite this, use remains limited, likely due to high costs.

In the absence of T2DM, semaglutide has been shown to improve cardiovascular outcomes, including reductions in MACE (HR 0.80; 95% CI 0.72–0.90) in people who are overweight (Body Mass Index ≥27 kg/m^2^) and have established CVD (Lincoff et al., 2023). However, it remains unclear whether the observed cardiovascular benefits in non-diabetic patients treated with semaglutide reflect a class effect.

While the exact mechanisms underlying this stroke risk reduction remain unclear, potential pathways may include effects on platelet aggregation, endothelial function and overall cardiometabolic health (Habib et al., 2020).

The close interrelationship among obesity, T2DM, CKD, HF, and CVD, together with accumulating evidence for the novel drug classes, has prompted the emergence of the concept of Cardiovascular–Kidney–Metabolic (CKM) syndrome and associated recommendations (Ndumele et al., 2026). Table 1 summarizes these recommendations and outlines the current indications for SGLT2 inhibitors and GLP-1 RA as stated in leading European and American clinical guidelines. These therapies are most appropriately considered in stroke survivors who meet guideline-supported indications, such as T2DM with high CV risk, HF, or CKD. Supplementary Table S1 provides an overview of the major trials conducted.

**Table 1:** Overview of contemporary guideline recommendations for the use of GLP-1 receptor agonists and SGLT2 inhibitors.

Guideline	GLP-1 RAs –recommendation	SGLT2 inhibitors–recommendation
ADA (American Diabetes Association) Standard of Care in Diabetes 2026 doi: 10.2337/dc26-S009	T2DM + ASCVD or indicators of high ASCVD risk: Recommended to use evidence–based GLP-1 RAs for CVD risk reduction and weight loss when appropriate.	T2DM + ASCVD: Recommended for CVD risk reduction.
2026 AHA/ACC/ADA/ASN Guideline for the Prevention, Detection, Evaluation, and Management of Cardiovascular-Kidney-Metabolic Syndrome doi: 10.1161/CIR.0000000000001453	T2DM + ASCVD: Recommended for CVD risk reduction (with proven CV benefit) (Class 1 recommendation, level of evidence A).	T2DM + ASCVD: Recommended for CVD risk reduction (Class 1 recommendation, level of evidence A).
	HF + CKM stage 4: Recommended in patients with obesity and symptomatic HFpEF (Class 1 recommendation, level of evidence A). Benefits in HFrEF are uncertain.	HF: Integral HF therapy (HFrEF; benefit across EF); recommended regardless of diabetes.
	CKD: not primary kidney-protective therapy.	CKD: Recommended to reduce MACE, CV death, and loss of kidney function in patients with urine ACR ≥200 mg/g and eGFR ≥20 ml/min per 1.73 m^2^ (Class 1 recommendation, level of evidence A).
2022 AHA (American heart association) Management of Heart Failure doi: 10.1161/CIR.0000000000001063	HF: Not recommended as HF-specific therapy	HF: Class 2a recommendation for HFmrEF and HFrEF, Class 1 recommendation, level of evidence A.
ESC (European Society of Cardiology) Diabetes 2023 doi: 10.1093/eurheartj/ehad192 Heart Failure guideline 2023 doi: 10.1093/eurheartj/ehad195	T2DM + ASCVD: Recommended for CVD risk reduction (Class 1 recommendation).	T2DM + ASCVD: Recommended for CVD risk reduction (Class 1 recommendation).
	HF: Not recommended as HF-specific therapy.	HF: Strongly recommended as core HF therapy (reduce HHF and CV events; applies irrespective of diabetes) (Class 1 recommendation, level of evidence A).
KDIGO 2024 CKD Guideline doi: 10.1016/j.kint.2023.10.018	CKD*:* not primary kidney-protective therapy for non-diabetic CKD.	CKD (with/without T2DM): recommend treating adults with CKD with an SGLT2 inhibitors for the following: eGFR ≥20 ml/min per 1.73 m2 and urine ACR ≥200 mg/g. (Class 1 recommendation, level of evidence A).
NICE (UK) guidelines Type 2 diabetes in adults: management, updated 18 February 2026	T2DM + ASCVD: First line therapy for its cardiovascular, renal and glycemic benefits.	T2DM + ASCVD: First line therapy for its cardiovascular, renal and glycemic benefits.

ACC, American College of Cardiolog; AHA, American heart association; ASN, American Society of Nephrolog; ASCVD, Atherosclerotic cardiovascular disease; ACR, Albumin-to-Creatinine Ratio; CKD, Chronic kidney disease; CKM, stage 4 is defined as clinical cardiovascular disease; ESC, European Society of Cardiology; HF, Heart failure; HHF, Hospitalization for heart failure; KDIGO, Kidney disease improving global outcomes; NICE, National Institute for Health and Care Excellence.

### In-hospital initiation and early follow-up

Both drug classes can, once the patient is clinically stable, usually be initiated during the hospital stay for acute stroke. SGLT2 inhibitors should be started after assessment of renal function and volume status, with counseling on the risk of genital infections, dehydration, and sick-day management. GLP-1 RA should be initiated at a low dose and titrated slowly to improve gastrointestinal tolerability. Always evaluate each patient individually to determine likely benefit. Early outpatient follow-up is recommended to assess adherence and tolerability, and to adjust concomitant medications as needed.

## Conclusion

The diagnostic work-up following an acute stroke should include screening for potentially modifiable risk factors, including T2DM, CKD, HF, and obesity, to reduce the risk of subsequent cardiovascular complications and events. When any of these conditions are identified, patients should be managed in accordance with current evidence-based guidelines, including consideration of SGLT2 inhibitors and/or GLP-1 RA when appropriate. Prior to initiating therapy, clinicians should carefully consider competing risks, particularly the substantial risk of all-cause mortality following stroke, alongside patient preferences. Clear pathways are also needed for responsibility, titration, and follow-up to ensure safe and effective use.

Additional studies are required to assess whether these agents reduce stroke risk (incidence, recurrence, or severity), and to identify the specific patient subgroups, care settings, and treatment contexts in which they confer benefit.

## Data Availability

The original contributions presented in the study are included in the article/Supplementary material, further inquiries can be directed to the corresponding authors.
