# Supplementary material for: Cardiometabolic drugs after stroke: time to embrace SGLT2 inhibitors and incretin-based therapies?
Source: Front Stroke. 2026 Aug 25;5:1918465. doi: 10.3389/fstro.2026.1918465 (PMC13549906; doi:10.3389/fstro.2026.1918465)
Supplement: Supplementary file 1 [file Data_Sheet_1.pdf]

Supplementary Table 1: Overview of the major trials conducted on SGLT2 inhibitors and GLP-1 RA.

Drug	Major trial(s) (Study population)	Approx. N	Main primary finding (effect sizes)	Prior stroke at baseline (approx.)	Stroke — overall (effect)
**SGLT2 inhibitors**					
**Empagliflozin**	EMPA-REG OUTCOME (T2DM)	~7,020	↓ 3-point MACE; large ↓ CV death (MACE HR ≈ 0.86); ↓ all-cause mortality	~23%	Neutral for stroke (no significant reduction)
	EMPEROR-Reduced (HFrEF;~50% with T2DM)	~3,730	↓ composite CV death or HHF (HR ≈ 0.75)	~11%	Stroke events infrequent; no clear difference
	EMPEROR-Preserved (HFpEF)	~5,988	↓ composite CV death or HHF (HR ≈ 0.79)	~10%	No significant effect on stroke reported
	EMPA-KIDNEY (CKD)	~6,600	↓ kidney disease progression & CV death (HR ≈ 0.72)	Not reported; 27% with CVD	No major stroke signal reported
**Dapagliflozin**	DECLARE-TIMI 58 (T2DM)	~17,160	No overall ↓ 3-point MACE; ↓ HHF and ↓ CV death or HHF composite	~8%	Neutral for stroke (no significant reduction)
	DAPA-HF (HFrEF)	~4,744	↓ worsening HF or CV death (HR ≈ 0.74)	~9%	Stroke events rare; no clear differences
	DELIVER (HFmrEF / HFpEF)	~6,263	↓ composite CV death or HHF (mainly fewer HHF) (HR ≈ 0.82)	~12%	No clear stroke effect reported
	DAPA-CKD (CKD)	~4,304	↓ composite renal outcome or CV death (HR ≈ 0.61)	~8%	No major stroke signal

**Canagliflozin**	CANVAS Program<br><br>(T2DM)	~10,142	↓ 3-point MACE (HR ≈ 0.86); safety signals: ↑ amputations, ↑ fractures (CANVAS)	~19%	Neutral for stroke across program
	CREDENCE<br><br>(T2DM + CKD)	~4,401	↓ composite renal outcome + CV death (HR ≈ 0.70)	~21%	No clear effect
**Ertugliflozin**	VERTIS-CV<br><br>(T2DM + ASCVD)	~8,246	Non-inferior for 3-point MACE; no significant MACE reduction	~23%	No significant stroke reduction (neutral)
**Sotagliflozin (dual SGLT1/2)**	SOLOIST-WHF<br><br>(T2DM + HHF)	~1,200 (stopped early)	↓ composite CV/HF events (HR ≈ 0.67) (stopped early)	Not reported	No major stroke signal reported
	SCORED<br><br>(T2DM + CKD)	~10,584 (stopped early)	↓ 3-point MACE (HR ≈ 0.84) (stopped early)	~9%	Significant reduction in stroke (HR ≈ 0.66)
**Other regional SGLT2s (ipragliflozin, luseogliflozin, tofogliflozin, remogliflozin, etc.)**	Various smaller/regional trials	varies	Focus on glycemic control/safety; limited large global CV/renal outcome data	generally low / not emphasized	Stroke data limited; no large outcome signals
**GLP-1 RA**					
**Liraglutide**	LEADER<br>(T2DM)	~9,340	↓ 3-point MACE; ↓ CV death & all-cause mortality	~16%	Neutral for stroke
**Semaglutide**	SUSTAIN-6 (subQ)<br>(T2DM)	~3,297	↓ 3-point MACE (HR ≈ 0.74)	~15%	Reduction in nonfatal stroke reported (HR ≈ 0.61)
**Semaglutide (oral)**	PIONEER-6<br>(T2DM)	~3,183	Cardiovascular safety; noninferior for MACE; underpowered for efficacy	~16%	Too few events for robust conclusions
**Dulaglutide**	REWIND<br>(T2DM)	~9,901	↓ 3-point MACE (HR ≈0.88)	Not reported; 32% with CVD	Significant reduction in stroke (HR ≈ 0.76)

**Exenatide ER**	EXSCEL<br>(T2DM)	~14,752	Non-inferior for MACE; neutral for efficacy	~17%	Neutral for stroke overall
**Albiglutide**	HARMONY Outcomes<br>(T2DM + CVD)	~9,463	↓ 3-point MACE (HR ≈ 0.78)	~18%	Neutral
**Efpeglenatide**	AMPLITUDE-O<br>(T2DM + CVD or CKD)	~4,076	↓ 3-point MACE (HR ≈ 0.73)	Not reported; 90% with CVD	Generally neutral to modest effects on stroke
**Tirzepatide (GIP/GLP-1)**	SURPASS CVOT<br>(T2DM + ASCVD)	~13,299	Non-inferior for MACE	~ 19%	Neutral for stroke
**Lixisenatide**	ELIXA<br>(T2DM + ACS)	~6,068	Neutral for MACE	~ 5%	Neutral for stroke

ACS Acute coronary syndrome, ASCVD Atherosclerotic cardiovascular disease, CKD Chronic kidney disease CV(D) Cardiovascular (disease), HF Heart failure, HFmrEF HF with moderate reduced EF, HFrEF HF with reduced ejection fraction, HFpEF, HF with preserved EF, HHF Hospitalization for heart failure HR Hazard ratio, MACE Major Adverse Cardiovascular Events, T2DM Type 2 Diabetes mellitus

The table is based on the following references:

SGLT2 inhibitors:

EMPA-REG OUTCOME — Zinman et al., New England Journal of Medicine, 2015.
EMPEROR-Reduced — Packer et al., New England Journal of Medicine, 2020.
EMPEROR-Preserved — Anker et al., New England Journal of Medicine, 2021.
EMPA-KIDNEY — (The EMPA-KIDNEY Collaborative Group,New England Journal of Medicine, 2023).
DECLARE-TIMI 58 (dapagliflozin) — Wiviott et al., New England Journal of Medicine, 2019.
DAPA-HF — McMurray et al., New England Journal of Medicine, 2019.
DELIVER — Solomon et al., New England Journal of Medicine, 2022.
DAPA-CKD — Heerspink et al., New England Journal of Medicine, 2020.
CANVAS Program (canagliflozin) — Neal et al., New England Journal of Medicine, 2017.
CREDENCE — Perkovic et al., New England Journal of Medicine, 2019.
VERTIS-CV (ertugliflozin) — Cannon et al., New England Journal of Medicine, 2020.
SOLOIST-WHF and SCORED (sotagliflozin; dual SGLT1/2) — Bhatt et al., Lancet (2020/2021)

GLP-1 receptor agonists

LEADER (liraglutide) — Marso et al., New England Journal of Medicine, 2016.
SUSTAIN-6 (subcutaneous semaglutide) — Marso et al., New England Journal of Medicine, 2016.
PIONEER-6 (oral semaglutide) — Husain et al., New England Journal of Medicine, 2019.
REWIND (dulaglutide) — Gerstein et al., Lancet, 2019.
EXSCEL (exenatide ER) — Holman et al., New England Journal of Medicine, 2017.
HARMONY Outcomes (albiglutide) — Hernandez et al., Lancet, 2018.
AMPLITUDE-O (efpeglenatide) — Gerstein et al., New England Journal of Medicine, 2021.
SURPASS CVOT (tirzepatide) — Nicholls et al. New England Journal of Medicine, 2025
ELIXA (lixisenatide) — Pfeffer et al., New England Journal of Medicine, 2015
